# 3′UTR-Mediated Gene Silencing of the Mixed Lineage Leukemia (MLL) Gene

**DOI:** 10.1371/journal.pone.0025449

**Published:** 2011-10-05

**Authors:** Maria Gomez-Benito, Fabricio Loayza-Puch, Joachim Oude Vrielink, Maria D. Odero, Reuven Agami

**Affiliations:** 1 Division of Gene Regulation The Netherlands Cancer Institute, Amsterdam, The Netherlands; 2 Center for Applied Medical Research, Pamplona, Spain; 3 Center for Biomedical Genetics, Utrecht, The Netherlands; Leiden University, The Netherlands

## Abstract

Translocations involving the Mixed Lineage Leukemia (MLL) gene generate in-frame fusions of MLL with more than 50 different partner genes (PGs). Common to all MLL translocations is the exchange not only of coding regions, but also of MLL and PG 3′-untranslated regions (3′UTRs). As a result, the MLL-PG fusion is normally highly expressed and considered the main driver of leukemia development, whereas the function of the PG-MLL fusions in leukemic disease is unclear. As 3′UTRs have been recognized as determinant regions for regulation of gene expression, we hypothesized that loss of the MLL 3′UTR could have a role in generating high MLL-PG levels and leukemia development. Here, we first tested the MLL-PG and PG-MLL mRNA levels in different leukemic cells and tumours and uncovered differential expression that indicates strong repression by the MLL-3′UTR. Reporter assays confirmed that the 3′UTR of MLL, but not of its main PGs, harbours a region that imposes a strong gene silencing effect. Gene suppression by the MLL 3′UTR was largely microRNA independent and did not affect mRNA stability, but inhibited transcription. This effect can at least partially be attributed to a tighter interaction of the MLL 3′UTR with RNA polymerase II than PG 3′UTRs, affecting its phosphorylation state. Altogether, our findings indicate that MLL translocations relieve oncogenic MLL-PG fusions from the repressive MLL 3′UTR, contributing to higher activity of these genes and leukaemia development.

## Introduction

**Figure 1:**
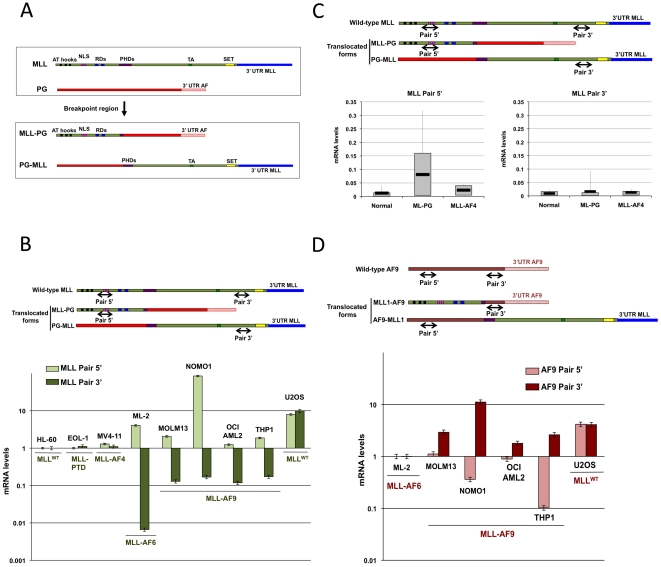
Expression pattern of MLL-PG and PG-MLL in leukemogenic cell lines and tumors. **A,** A schematic representation of MLL translocations with a partner gene (PG) at the mRNA level. AT hooks are designated (AT hooks domain); NLS (Nuclear Localization Signal); RDs (Repressor domains); PHDs (Plant homology domains); TA (Taspase domains); SET (methyl-transferase domain). **B,** RT-qPCR analyses, with 5′and 3′pair primers, as depicted, to detect MLL, were performed with RNA extracted from several cell lines, as indicated. The status of MLL is annotated and summarized in Supplementary Table S1. **C,** MLL mRNA quantification using the same primer sets as above of RNA extracted from leukemogenic patient samples with or without MLL-translocations. The status of MLL is annotated and summarized in Supplementary Table S2. Patients mRNA levels expression was evaluated individually but statistically analized as pulls regarding MLL status. **D,** RT-qPCR to quantify AF-9 mRNA levels by using the indicated 3′ and 5′ primers in cell lines harboring MLL translocations as indicated. The status of MLL is annotated and summarized in Supplementary Table S1.

The mixed lineage leukemia (MLL) proto-oncongene is a recurrent site of genomic rearrangements in acute myeloid leukemias (AML) [1]. Uniquely, MLL is rearranged with more than 50 different partner genes (PGs), all involving the in-frame fusion of MLL 5′-terminal part to PG (MLL-PG) and a reciprocal fusion of PG to MLL 3′-terminal part (PG-MLL) [1], [2] (Fig. 1A).

Since the discovery of MLL in 1992 [3], many advances have been made in understanding its role in leukemia [1], [4], [5], [6]. Five MLL translocations account for approximately 80% of MLL rearrangements: MLL-AF4 [t(4;11)(q21;q23)], MLL-AF6 [t(6;11)(q27;q23)], MLL-AF9 [t(9;11)(p22;q23)], MLL-ELL [t(11;19)(q23;p13.1)] and MLL-ENL [t(11;19)(q23;p13.3)] [7], [8], although more than 70 different translocations have been identified [8]. Intriguingly, all translocations involving MLL generate two common different events: a gain of function (MLL-PG), which is normally highly expressed, and haplo-insufficiency for wild-type MLL [4], [9]. Furthermore, current evidence indicates that formation of dimers of MLL N-terminal domains might be important for leukemogenesis [10], and this could be favoured after MLL translocation and MLL-PG overexpression. Certainly, as all MLL-PG fusions, regardless of the PG, exhibit a distinct gene expression signature and induce acute leukemia development [2], [11], the MLL-PG overexpression and dimerization may play a role.

3′UTRs have been recognized as major regulatory regions in genes [12], [13]. They can mediate gene regulation mainly at the level of translation and mRNA stability by providing docking sites for microRNAs [13], [14] and RNA binding proteins [15]. Changes in 3′UTR length, sequence or location can influence gene expression, cell proliferation and survival and lead to cancer development. For instance, translocations affecting the oncogene HMGA2 swap its 3′UTR for that of another gene resulting in loss of the let-7 miRNA target sites, escaping from miRNA-mediated gene repression and leading to HMGA2 overexpression and tumorigenesis [16]. Moreover, many genes contain proximal and distal polyadenylation signals (PASs) in their 3′UTRs, that when aberrantly activated will lead to shortening of the 3′UTR and escape of miRNAs or RBPs repression as found in cyclin D1 and D2 [12]. Nonetheless, apart from 3′UTR shortening, translocation or PAS mutation, other 3′UTR-mediated gene expression control mechanisms at the RNA level may exist and still remain to be identified.

Here, we have investigated the role of MLL-3′UTR in restricting MLL expression. Our results suggest that by exchanging 3′UTRs, MLL-PG translocations evade a strong transcriptional gene repressive control mediated by the 3′UTR of MLL which leads to MLL-PG overexpression and leukemogenesis.

## Results

MLL translocations generate two different chimeric genes: MLL-PG and PG-MLL (Fig. 1A). To address if MLL-PG and PG-MLL expression differ at the mRNA level, we designed two different pair of primers (Fig. 1B). The 5′pair measures wt MLL and MLL-PG mRNA levels while the 3′pair measures wt MLL and PG-MLL mRNA levels. In the absence of MLL translocation, equal mRNA levels must be found for both 5′ and 3′pair primers. Indeed, the cell lines HL-60 and U2OS that are known to have wild-type (wt) MLL and the EOL cell line, which contains partial tandem duplication (MLL-PTD), showed no difference between both primer pairs quantification (Fig. 1B). We therefore used these cell lines to normalize MLL expression in the leukemic cell lines with MLL translocations. We expected that if the translocations affected the relative abundance of MLL-PG mRNA with regard to PG-MLL, an inverse change in the level of mRNA detected by the 5′pair and the 3′ pair should be observed in cell lines harboring MLL rearrangements. Intriguingly, all cell lines with MLL-AF9 and MLL-AF6 translocations showed a relatively higher expression of mRNA detected by the 5′ pair, while a marked reduction by the 3′pair (Fig. 1B). Only the cell line MV4-11 with an MLL-AF4 translocation showed similar 5′ and 3′ mRNA expression (Fig. 1B), suggesting that in this case no relative change in expression was induced by the exchange of MLL and AF4 3′UTRs. In addition to cell lines, we performed q-RT-PCR quantification of 5′ and 3′MLL expression in AML patient samples. The type of translocation was determined by FISH analysis (see methods). A consistent pattern to the cell lines was revealed: patients with no MLL translocations showed no difference in 5′ and 3′ detection primers, patients harboring MLL-PG translocations (excluding MLL-AF4) presented a higher 5′MLL expression, pointing to a higher MLL-PG expression in relation to PG-MLL mRNA levels, and patients with MLL-AF4 translocation showed no statically significant differences in mRNA levels between both primer pairs (Fig. 1C). All together, these results demonstrate that MLL translocations, with the exception of AF4, lead to higher levels MLL-PG and inhibition of PG-MLL expression.

The change in relative PG-MLL expression observed upon translocation could be explained by low activity of PG promoter, or by repression inflicted by MLL 3′UTR region, or both. To distinguish between these possibilities we measured the expression of PG-AF9 in MLL-AF9 cell lines using 5′ and 3′ PCR primer pair sets designed in the same way we did for MLL mRNA levels (Fig. 1D). We used ML-2 cell line (harboring an MLL-AF6 translocation) to normalize the data. First, both primer sets identified consistent AF9 mRNA expression in all cell lines with wt AF9, indicating that AF-9 promoter is active (data not shown). Second, in all the cell lines with MLL-AF9 translocations a relatively higher expression level of 3′ AF9 mRNA (MLL-AF9) was observed, accompanied with either a reduction, or no change, in 5′-AF9 (AF9-MLL) expression (Fig. 1D). This antagonistic pattern of change in expression for both MLL and AF9 in MLL-AF9 translocations excludes the possibility of AF9-MLL silencing due to AF9 low promoter activity, while pinpointing an active repression of AF9-MLL expression by MLL's 3′ region.

**Figure 2:**
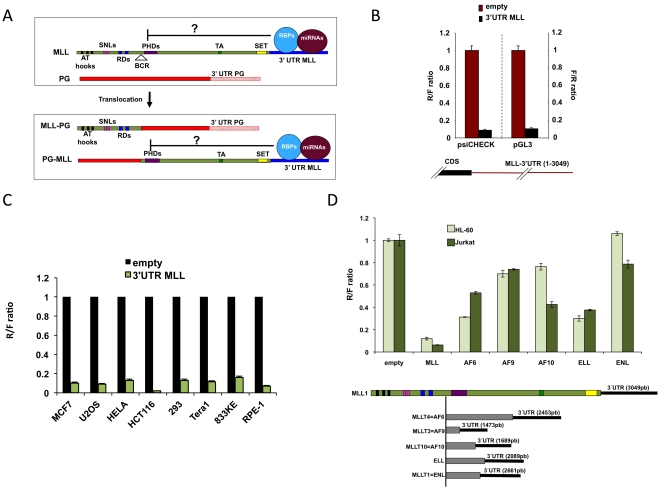
MLL-3′UTR strongly inhibits gene expression. **A,** A schematic representation of MLL translocations at the mRNA level with putative RBPs and miRNAs binding to MLL-3′UTR and affecting gene expression. (PG). **B,** Transient transfection experiments in MCF-7 cells (MLL wt) using Ψ2, a dual luciferase vector (Fig. S1), and pGL3 luciferase reporter constructs, with or without the human MLL-3′UTR. Together with the pGL3 constructs, we transfected pLR-SV40 (*Renilla*) as control. Relative *Renilla* and *Firefly* ratios are presented (R/F or F/R) as indicated. **C,** Ψ2 constructs, with or without MLL-3′UTR, were transiently transfected into the indicated cell lines and the relative R/F ratio was calculated. **D,** Transient transfection of Ψ2 reporter constructs containing the 3′UTRs of MLL or its main PGs in HL-60 and Jurkat leukemic cell lines (both MLL wt). In the lower part a graphic comparison of the different 3′UTRs length. Coding region is depicted in grey and 3′UTRs in black.

To examine whether the MLL-3′UTR can potently suppress gene expression (Fig. 2A) we cloned it downstream of the *Renilla* luciferase reporter gene in the psiCheck2 (Ψ2) dual reporter vector (where *Renilla* luciferase and control *Firefly* luciferase are expressed from the same vector) (Fig. S1A), and downstream of the *Firefly* luciferase gene in the pGL3 vector. In both cases, strong repression (∼10 folds) was mediated by MLL-3′UTR (Fig. 2B). This suppression was seen in all cell lines tested thus far (Fig. 2C), regardless of their tissue of origin, including HL-60 and Jurkat leukemic cell lines (Fig. 2D), and was also observed when examining the 3′UTR of mouse MLL (Fig. S1B). Most interestingly, the main PGs-3′UTRs [8] had only a mild gene reporter suppressive effect or no effect at all, when analyzed in different cell lines (Figs. 2D and S1C). As MLL-3′UTR length is not significantly different from that of its PGs (Fig. 2D), we suggest that MLL-3′UTR could possess strong gene suppressive information.

**Figure 3:**
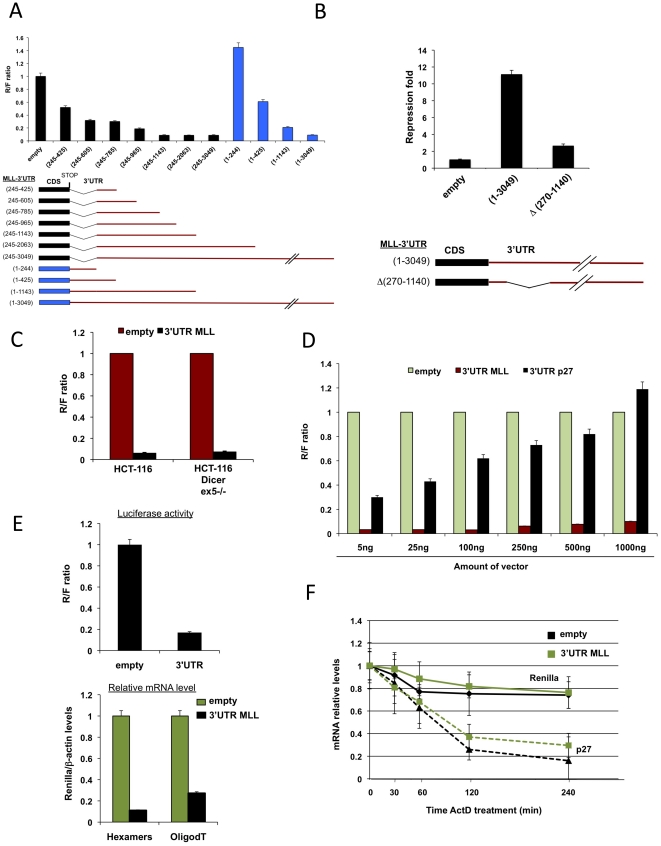
Inhibition of gene expression by MLL-3′UTR is at the mRNA level but largely independent of miRNAs and mRNA stability. **A and B,** Several regions of MLL-3′UTR, as depicted, were cloned downstream of *Renilla* in the Ψ2 vector, then transfected to U2OS cells and luciferase activity was measured. The black thin lines represent deletion regions. **C,** Ψ2 constructs, with or without MLL-3′UTR, were transiently transfected into the indicated cell lines and *Renilla* activity relative to *Firefly* activity was measured. **D,** Transient transfection of increasing amounts of Ψ2 empty, MLL-3′UTR or p27-3′UTR reporter constructs into U2OS cells. Relative R/F is presented. **E,** U2OS cells were transiently transfected with Ψ2 empty and MLL-3′UTR. 48 h later cells were harvested and both luciferase activity as well as mRNA levels of *Renilla* and b-Actin were determined. Presented are the relative R/F activity ratios and the *Renilla*/b-Actin mRNA ratios from the same cell population. **F,** U2OS cells were transiently transfected with Ψ2 empty and MLL-3′UTR reporter constructs. Forty eight hours later, actinomycin D (ActD) was added and cells were harvested at the indicated time points thereafter. Then, RNA was extracted and levels of *Renilla*, b-Actin and p27 mRNAs were determined. Presented are the *Renilla* and p27 mRNA levels relative to b-Actin and to time point 0.

To further analyze MLL-3′UTR, we dissected the 3 kb long MLL-3′UTR into consecutive smaller regions. Figure 3A shows that the region between nucleotides (nt) 245–1143 constitutes the smallest region conferring the highest repression, while the 1–245 region had a slight positive effect. Shorter fragments within the 245–1143 region had a significant but more moderate effect, indicating that repression was not localized to one particular sequence motif but rather was dispersed along the whole region. In accordance, excising the 270—1140 region out of the entire MLL-3′UTR relieved the bulk of the repression effect (Fig. 3B), indicating that most of the inhibitory effect on gene expression is indeed localized to this ∼900 bp region.

miRNAs are known to mediate repression of gene expression by sequence specific interaction with 3′UTRs to elicit induction of mRNA instability and inhibition of protein translation [13], [17], [18]. Therefore, we examined whether they could be responsible for MLL-3′UTR suppression. Although no conserved miRNAs have been predicted in the region (245–1143) nt of MLL-3′UTR, we assessed its effect on *Renilla* gene expression in HCT-116-Dicer ex5^−/−^ (which contains a deletion in exon 5 of Dicer, a key player in the generation of miRNAs [19]) compared with parental HCT-116 cells (Fig. 3C). No difference in *Renilla* activity was found between these two cell lines (Fig. 3C), suggesting that miRNAs play a minor role in this phenotype. To strengthen this result we evaluated the effect of increasing MLL-3′UTR levels on reporter expression and compared it with the increase of p27-3′UTR in a miR-221 expressing cell line [14], as it has been shown that miRNA activity can be sequestered by artificially increasing the cellular amount of target 3′UTR mRNA [20]. While miR-221 effect over p27-3′UTR was saturated by target 3′UTR level increase, MLL-3′UTR suppressive effect was only mildly reduced by the increase in transfected reporter plasmid (Fig. 3D). Altogether, miRNAs seemed unlikely to be responsible for MLL-3′UTR-mediated repression.

Next, we investigated the mechanism of MLL-3′UTR-mediated gene repression by examining mRNA level and stability. We transfected cells with Ψ2-empty or Ψ2-MLL-3′UTR and examined *Renilla* and *Firefly* luciferase activity (protein levels) and mRNA levels. Similar reduction in *Renilla* activity and *Renilla* mRNA levels in the presence of MLL-3′UTR were found with respect to normalizer *Firefly*, suggesting that MLL-3′UTR repression was mediated by markedly reducing mRNA levels (Fig. 3E). We then measured the stability of *Renilla* mRNA using actinomycin D (ActD), an inhibitor of RNA Polymerase II (RNAPII) [21]. While the *Renilla* mRNA level was highly reduced when MLL-3′UTR was present (Fig. 3E), its stability remained high (Fig. 3F). We controlled ActD by measuring the labile p27 mRNA (half life ∼1 hour [22]. These results suggest that MLL-3′UTR may affect mRNA synthesis.

**Figure 4:**
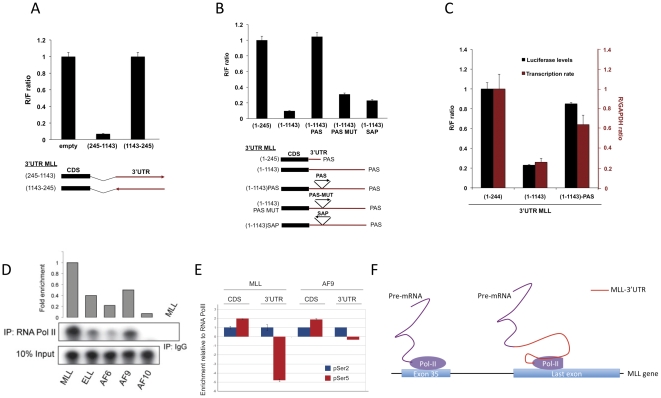
The 3′UTR of MLL inhibits mRNA synthesis. **A and B,** The indicated Ψ2 constructs were transfected to U2OS cells and *Renilla* and *Firefly* luciferase was measured to calculate R/F ratios. PAS is the polyadenylation signal region of SV40 (PAS); PAS mutant (PAS-MUT) has four nucleotide alterations in the PAS consensus motif; (SAP) is the PAS region in a reverse orientation (See Supplementary Material). All PAS regions were cloned at position 245 of MLL-3′UTR. **C,** U2OS cells were transfected with the indicated Ψ2 reporter constructs. Forty-eight hours later, 4-thiouridine (4SU) was added and cells were incubated for 4 hours to mark newly transcribed RNA. Subsequently, RNA was isolated and the newly transcribed mRNA was purified as described ([22], see material and methods). Relative levels of *Renilla* mRNA (to GAPDH) were determined and compared to *Renilla/Firefly* luciferase activity extracted from the same cell population. **D,** RNAPII was IPed from U2OS cells and incubated with RNA probes containing MLL (245–1143)-3′UTR, or PG-3′UTRs. Following incubation, beads were washed, RNA extracted and loaded on to a denaturing gel. Right upper panel shows an immunoblot analysis with a RNAPII antibody. This experiment was repeated 3 times with a similar result. **E,** U2OS cells were transfected with Ψ2-MLL-3′UTR or Ψ2-AF9-3′UTR and subjected to ChIP analysis with an antibody directed against the carboxy-ternminal region (CTD) of RNAPII, and the CTD phosphor-specific antibodies phospho-S2 and S5. Bound *Renilla* (CDS) and 3′UTR regions in the ChIP were detected by PCR, and the enrichment of S2 and S5 compared to RNAPII in IPs is shown. F, A schematic representation of MLL-3′UTR interaction with RNAPII and its speculative effect on rate of transcription.

To examine the effect of MLL-3′UTR on mRNA synthesis, we either flipped MLL-3′UTR 3′-5′ (Fig. 4A), or inserted a wt polyadenylation signal and cleavage site (PAS), at position 245 (upstream of the repression region) of MLL-3′UTR (Fig. 4B). 3′end sequencing analysis showed the expected cleavage and polyadenylation site triggered by this PAS (Supplementary Fig. S1D). Interestingly, both manipulations resulted in complete abolishment of MLL-3′UTR repression. As controls we used a mutant PAS (PAS-MUT), and a PAS constructed in a reverse orientation (SAP), both hardly affected MLL-3′UTR repression (Fig. 4B). These results indicate that MLL-3′UTR influence transcription, only when it is part of the transcribed unit. To directly examine this possibility, we measured *in vivo* transcription rates of *Renilla* and control GAPDH mRNAs using 4-thiouridine (4SU, [22], see materials and methods). Figure 4C shows that MLL-3′UTR had a major effect on transcription rate, which was comparable to the reduction seen with *Renilla* luciferase activity from the same experiment. Moreover, the inclusion of a PAS prior to the repression region (same construct presented in Fig. 4B) relieved most of transcription repression and, as expected, *Renilla* luciferase activity.

We then hypothesize that RNA synthesis could be blocked by RNA polymerase II (RNAPII) engaging and halting while transcribing MLL-3′UTR. To test this hypothesis, we IPed RNAPII from U2OS cells and incubated beads with either MLL-3′UTR or the main PGs-3′UTRs RNA probes. RNAPII IP was verified by an immunoblot analysis (Fig. 4D) and IgG IP was used to monitor specificity of RNAPII binding to 3′UTRs. Figure 4D shows a stronger retention of MLL-3′UTR by RNAPII (2 to 10 times) compared to the 3′UTRs of the PGs. Therefore, these results demonstrate an exclusively stronger RNAPII interaction with MLL-3′UTR that could correlate with a RNAPII halt in transcription inside the 3′UTR (Fig. 4E). To further characterize this interaction we performed chromatin IPs (ChIPs) with antibodies directed against phosphor-serine (pSer) 2, 5, or an antibody against all forms of the carboxy-terminal domain of RNAPII (general), and calculated enrichment values of the phosphorylated forms over the general. We found that inclusion of MLL-3′UTR causes a sharp loss of pSer5, while pSer2 remained unchanged compared to AF-9-3′UTR, which was used as control (Fig. 4E). No difference was detected in the coding sequence (CDS). While this result is correlative in its nature, it has the potential to explain, at least in part, the gene expression repressive effect of 3′-UTR on MLL and the overexpression of MLL-PG form upon MLL translocations.

## Discussion

3′UTRs are mainly viewed as regulators of mRNA stability and translation through the binding of miRNAs or RNA-binding proteins (RBPs). Here, we observed that 3′UTRs could also influence gene transcription. The 3′UTR of MLL, when placed downstream of a reporter gene, and when was part of the transcribed unit, led to a dramatic reduction in gene expression levels. This effect did not map to a particular motif, but it was rather smeared along a large region of 1 kb in MLL-3′UTR. On the contrary, a similar pattern on reporter gene expression was not observed with the 3′UTRs of main MLL partner genes (PGs). Interestingly, the repression mediated by MLL-3′UTR could be avoided by a preceding wt polyadenylation and cleavage site (PAS), but not by a mutated PAS or by increasing the copy number of 3′UTR units in the cells. Furthermore, MLL-3′UTR, but not PGs-3′UTRs, showed enhanced interaction with RNAPII, suggesting a model by which the newly synthesized MLL-3′UTR RNA interferes with RNAPII progression (Fig. 4F). How exactly this mRNA region affects RNAPII activity is not clear at the moment. It is possible that, once synthesized, MLL-3′UTR either modifies RNAPII conformation to become a less efficient enzyme, displace essential factors, or alternatively recruits RNA-binding proteins that affect RNAPII activity. Alternatively, the MLL-3′UTR region may affect initiation of transcription. One hint we found is the correlation of MLL-3′UTR with a rapid loss of pSer5 from RNAPII-CTD. How exactly this causes a change in RNAPII activity, and which factors are involved, remain to be elucidated. Nevertheless, MLL-3′UTR acts to restrict MLL expression and its loss by translocation could be an optimal way to assure a higher rate of N-terminal MLL domains transcription, in the form of MLL-PG mRNA. High expression of N-MLL would facilitate MLL-dimerization and would promote leukemogenic transformation [23].

## Materials and Methods

### Cell lines and patient samples

Jurkat, HL-60, EOL-1, MV4-11, ML-2, MOLM13, NOMO1, OCI-AML2 and THP-1 were cultured in RPMI1640 and U2OS, HeLa, HEK293, MCF7, HCT116, and HCT116 Dicer ex5−/− in DMEM all supplemented with 10% fetal calf serum and antibiotics. Cell lines were obtained from ATCC or DSMZ, except HCT116 and HCT116 Dicer ex5−/− that were a kind gift of Dr. Bert Vogelstein). Molecular characteristics of cell lines regarding MLL are summarized in Table S1.

Patient samples were collected at the Department of Genetics, University of Navarra, Spain. Leukemic blasts were obtained from bone marrow (BM) of AML patients with more than 60% blasts. The study has been approved by the Ethics Committee for Research with Human Subjects and was carried out in accordance with the ethical guidelines of our institution. Clinical and molecular characteristics of the patients are summarized in Table S2.

FISH analysis were performed as previously described [24] using a dual color locus specific MLL probe (Vysis, Downers Grove, IL).

### Luciferase Constructs

For details see Data S1.

### Luciferase Activity Analysis

For luciferase analysis, suspension cells were nucleofected (amaxa technologies), while adherent cells were transfected using Fugene. Dual luciferase-activity assays were performed 48 h after transfection according to the manufacturer's directions (Promega). Results are represented as means and standard deviation (SD) from three independent experiments.

### qRT-PCR Analysis

Total RNA was extracted using TRIzol reagent (Life Technologies) according to the manufacturer's instructions. Synthesis of cDNA with Superscript III reverse transcriptase (Invitrogen) was primed with oligo(dT). Primers (Data S1) were designed to amplify 150–200 bp fragments. Analyses were carried out using SYBR Green PCR master mix (Applied Biosystems) and the ABI Prism 7000 system (Amersham-Pharmacia). The results were normalized with respect to human β-actin. Ct values for gene expression were calculated according to the Ct method.

### Run-on

U2OS cells were transfected by means of Fugene with psi-CHEk-2- empty, psi-CHECk-2- MLL-3′UTR (1–1143) or the same construct with the PAS at position 245. Forty hours later cells were incubated with 500 uM 4-Thio-Uridine (4sU, Sigma) for 4 hr prior to RNA extraction by the Trizol method. Biotinylation of 4sU-labeled RNA was performed using EZ-Link-Biotin-HPDP (Pierce) dissolved in DMSO. Biotinylation of 100 ug total RNA was carried out in 10 mM Tris (pH 7.4), 1 mM EDTA and 0.2 mg/ml Biotin-HPDP at a final RNA concentration of 100 ng/ul for 1.5 h at room temperature. Unbound Biotin-HPDP was efficiently removed by chloroform/isoamylalcohol (24∶1) extraction. Afterward, RNA was precipitated by addition of a 0.1 volume of 5 M NaCl and an equal volume of isopropanol, and then washed with 70% ethanol. The RNA were resuspended in RNase-free water and treated with DNase I for 1 h at 37°C and 65°C for 10 min to inactivate the enzyme and denaturate the RNA. Denaturated RNA was then incubated with Dynabeads M-280 Streptavidin (Invitrogen) in rotation for 30 min at room temperature. Beads were transferred and magnetically fixed to columns. In the columns the beads were wash with washing buffer (100 mM Tris pH 7.4, 10 mM EDTA, 1 M NaCl,0.1% Tween20) and eluted by the addition of 100 mM dithiothreitol (DTT). Biotinylated RNA was recovered by the eluted fraction using the RNeasy Mini Elute Spin Columns (Qiagen) and was subjected to cDNA synthesis with oligo(dT) and qRT-PCR analysis using Renilla and GAPDH primers.

### RNAPII IP and binding to RNA probes

U2OS cells were lysed in immunoprecipitation buffer (50 mM Tris [pH 8.0], 150 mM NaCl, 1 mM NaF, 1% NP-40 supplemented with protease inhibitors). Lysates were centrifuged for 10 min at 13,000× g and the supernatant was incubated for 1 hr with magnetic beads (Invitrogen) coated with anti-RNA Polymerase II (Upstate). The beads were washed four times with inmunoprecipitation buffer and two times with RNA binding buffer (50 mM Tris-HCl [pH 8.0], 10% glycerol, 0.2 mg/ml BSA, 0.01% NP-40, 0.5 mM DTT, 100 mM KCl, 2 mM MgCl2). After the last wash, the beads were resuspended in 200 µl of RNA binding buffer and incubated with 15,000 cpm of radiolabeled in vitro transcribed RNA (Ambion) for 15 min at room temperature. The beads were washed three times with RNA binding buffer. Beads were suspended in 20 µl of water with 1% SDS and 200 µg/ml of Proteinase K and incubated for 30 min at 50 C. The whole volume was applied to a denaturing polyacrylamide gel (3%). The radioactive retained RNA was quantified using a Fuji Phosphoimager BAS 100.

### ChIP

U2OS cells were transfected with either 100 ng of Ψ2-MLL-3′UTR or 100 ng of Ψ2-AF9-3′UTR and grown in DMEM (10% FCS) for 48 hours, crosslinked with 1% formaledehyde for 10 minutes and the crosslinking was stopped with 0.125 M glycine for 5 minutes at room temperature. Cells were then washed with ice cold 1× PBS. Cell pellets were resuspended and incubated in cell lysis buffer (50 mM Tris-HCl [pH 8.0], 85 mM KCl, 0.5% NP40, and protease inhibitor (Roche)) for 10 minutes. Nuclear pellets were spun down at 5,000 rpm for 5 minutes, resuspended in nuclear lysis buffer (1% SDS, 10 mM EDTA, 50 mM Tris⋅HCl (pH 8.0)), and incubated for another 10 minutes. Chromatin was sonicated for 30 minutes, with pulses every 30 seconds and then centrifuged at 14,000 rpm for 10 minutes to remove the debris. 5 mg of anti-RNA Polymerase II (Millipore), anti pSer2-Polymerase II, or anti pSer5-polymerase II (Covance) was added to the lysates and incubated at 4°C overnight. 40 mg of agarose protein A was added and incubated for 1 hour at 4°C. Beads were washed 5 times. The DNA complexes were eluted and reverse crosslinked in 0.3 M NaCl at 65°C for 4 hours. Protein was digested with proteinase K at 45°C for 1 hour and the DNA was purified with Qiagen PCR columns. To show differential enrichment of phosphorilated forms of RNA Polymerase II to either Renilla CDS or MLL and AF9 3′UTR, the purified DNA was amplified with following primers:

Renilla Forward CGGAAACTGGAGCCTGAGGA

Renilla Reverse AACCCAGGGTCGGACTCGAT

MLL 3′UTR Forward ATGGGGTCCCTAGCAGACTT

MLL 3′UTR Reverse TTCGACAGACGCTGTAGGTG

AF9 3′UTR Forward GACAGCTCAACAATGCTGGA

AF9 3′UTR Reverse GGACCAAATAGCCACCTTGA

P7-T25N CAAGCAGAAGACGGCATACGAGATTTTTTTTTTTTTTTTTTTTTTTTTVN

P7 CAAGCAGAAGACGGCATACGAGAT

A written consent was obtained from all participants involved in this study.

## Supporting Information

Data S1 Raw Ct for AF9 mRNA levels quantification with the 5′and 3′pairs, respectively. B, Oligos used for constructs making, indicating length and restriction enzymes used. C, Oligos sed for quantitative Real-Time PCR of the indicated genes.
(PDF)

Figure S1 **A**, a schematic drawing of the psiCHECK reporter vector backbone used in this study. **B**, transient transfection of psiCHECK reporter constructs containing human and mouse MLL-3′UTRs in MCF-7 cells. **C**, transient transfection of psiCHECK reporter constructs containing MLL and main PG 3′UTRs. D, Cells were transfected with Ψ2-PAS-MLL-3′UTR and 3′end analysis was performed using RT with a P7-T25N oligo and PCR with P7 and a sequence specific primer. Sequence analysis of the PCR product shows the correct cleavage and polyadenylation induced by the SV40-PAS.
(JPG)

Table S1 Main clinical and molecular characteristics of cells lines used in MLL-PG and PG-MLL mRNA levels evaluation. MLL-PTD means MLL partial tandem duplication, AML-M2, AML-M4 and MLL-M5 refer to different subtypes of AML according to the French-American British (FAB) classification. T-ALL refers to T cell lynphocytic leukemia.
(DOCX)

Table S2 Main clinical and molecular characteristics of patient samples are shown. Sex (M means male; F means female), AML-M2, AML-M4 and MLL-M5 refer to different subtypes of AML according to the French-American British (FAB) classification. ALL refers to acute lymphocytic leukemia and MDS to myelodysplastic syndrome.
(DOCX)
