# Correction: 3′UTR-Mediated Gene Silencing of the Mixed Lineage Leukemia (MLL) Gene

**DOI:** 10.1371/annotation/d39cb963-9277-462f-8070-6409d6fcf484

**Published:** 2011-11-10

**Authors:** Maria Gomez-Benito, Fabricio Loayza-Puch, Joachim A. F. Oude Vrielink, Maria D. Odero, Reuven Agami

The correct third author name is: Joachim A. F. Oude Vrielink.

The correct citation is: Gomez-Benito M, Loayza-Puch F, Oude Vrielink JAF, Odero MD, Agami R (2011) 3′UTR-Mediated Gene Silencing of the Mixed Lineage Leukemia (MLL) Gene. PLoS ONE 6(10): e25449. doi:10.1371/journal.pone.0025449

The correct author contributions are: Conceived and designed the experiments: MG-B RA. Performed the experiments: MG-B FL-P JAFOV. Analyzed the data: MG-B FL-P RA. Contributed reagents/materials/analysis tools: MDO. Wrote the paper: MG-B RA. 

