# Supplementary material for: 3′UTR-Mediated Gene Silencing of the Mixed Lineage Leukemia (MLL) Gene
Source: PLoS One. 2011 Oct 5;6(10):e25449. doi: 10.1371/journal.pone.0025449 (PMC3187771; doi:10.1371/journal.pone.0025449)
Supplement: Data S1 — Raw Ct for AF9 mRNA levels quantification with the 5′and 3′pairs, respectively. B, Oligos used for constructs making, indicating length and restriction enzymes used. C, Oligos sed for quantitative Real-Time PCR of the indicated genes. (PDF) [file pone.0025449.s001.pdf]

	AF-9 N-t	AF-9 C-t	b-actin
EOL-1	24.33	21.69	16.31
	24.13	21.87	16.48
	24.22	21.74	16.38
	24.22666667	21.76666667	16.39
	7.836666667	5.376666667	
ML-2	28.34	28.32	17.34
	28.53	28.33	17.66
	28.14	28.09	17.77
	28.33666667	28.24666667	17.59
	10.74666667	10.65666667	
MOLM13	29.31	27.79	18.79
	29.05	28.02	18.25
	29.22	27.32	18.77
	29.19333333	27.71	18.60333333
	10.59333333	9.11	
NOMO1	30.66	32.47	18.09
	30.71	32.81	18.56
	30.72	32.7	18.81
	30.69666667	32.66	18.48666667
	5.216666667	7.18	
OCI	27.67	26.5	16.34
	27.44	26.43	16.67
	27.41	26.3	16.76
	27.50666667	26.41	16.59
	10.91666667	9.82	
THP1	31.95	27.38	18.59
	32.69	27.66	17.82
	32.17	27.47	18.29
	32.27	27.50333333	18.23333333
	14.04	9.273333333	
U2OS	25.77	25.98	17.08
	25.86	25.81	17.37
	26.07	25.71	17.18
	25.9	25.83333333	17.21
	8.69	8.623333333	

	PCR cloning	
	**Forward**	**Reverse**
**psiCHECk-2 (PmeI-NotI)**		
**Human MLL 3' UTR**		
**(1-3049)**	CGCC**GATATC**AGCTGCTCTTCTCCCCCAGTGTTGGA (EcoRV)	GCCG**GCGGCCGC**TGGGGGATTCTTGGGAATGACCCATC (NotI)
**(1-245)**	CGCC**GATATC**AGCTGCTCTTCTCCCCCAGTGTTGGA (EcoRV)	GCCG**GCGGCCGC**AAACAATTGGAGGGGCCCCAGGAGAA (NotI)
**(245-425)**	CGGC**GATATC**ACTGTTAGAAAGTGGGAATGGGGTCC (EcoRV)	GCCG**GCGGCCGC**ACCCTCCATTTGGGGCTTTGCCTGAC (NotI)
**(245-605)**	CGGC**GATATC**ACTGTTAGAAAGTGGGAATGGGGTCC (EcoRV)	GCCG**GCGGCCGC**TGGTAATTAATTGGCACAACCCCATG (NotI)
**(245-785)**	CGGC**GATATC**ACTGTTAGAAAGTGGGAATGGGGTCC (EcoRV)	GCCG**GCGGCCGC**TCCCCTACAACCCGAAAGAAGCA (NotI)
**(245-965)**	CGGC**GATATC**ACTGTTAGAAAGTGGGAATGGGGTCC (EcoRV)	GCCG**GCGGCCGC**CATGCTACATATGTTTCTTTAAAACT (Not I)
**(245-1143)**	CGGC**GATATC**ACTGTTAGAAAGTGGGAATGGGGTCC (EcoRV)	GCCG**GCGGCCGC**TGTTCTGCCTTCAACTGGACACTGAC (NotI)
**(245-2063)**	CGGC**GATATC**ACTGTTAGAAAGTGGGAATGGGGTCC (EcoRV)	GCCG**GCGGCCGC**ATCTGCTTTATCGTGAGTGGTCAGAC (NotI)
**(1-425)**	CGCC**GATATC**AGCTGCTCTTCTCCCCCAGTGTTGGA (EcoRV)	GCCG**GCGGCCGC**ACCCTCCATTTGGGGCTTTGCCTGAC (NotI)
**(1-1143)**	CGCC**GATATC**AGCTGCTCTTCTCCCCCAGTGTTGGA (EcoRV)	GCCG**GCGGCCGC**TGTTCTGCCTTCAACTGGACACTGAC (NotI)
**(1143-245)**	GCCG**GCGGCCGC**ACTGTTAGAAAGTGGGAATGGGGTCC (NotI)	CGGC**GATATC**TGTTCTGCCTTCAACTGGACACTGAC (EcoRV)
**AF6 3' UTR**	CGGC**GATATC**GGCAGCTAGAATTTTACCAAGTTAGC (EcoRV)	GCCG**GCGGCCGC**AACAGCAACAGCAACAAGATATTCAA (NotI)
**AF9 3' UTR**	CGGC**GATATC**CAACTGGATGCATCAAGAACTATTGTG (EcoRV)	GCCG**GCGGCCGC**GAATTTCTAGTAAAAACTTTGAAGATG (NotI)
**AF10 3' UTR**	CGGC**GATATC**CAAGACTTAGTGATAAAACTGGGCCT (EcoRV)	GCCG**GCGGCCGC**AATTTTATTAACAAAACATTTTGTAC (NotI)
**ELL 3' UTR**	CGGC**GATATC**CCGCCCTCCCCGATGGCGGGGATCTG (EcoRV)	GCCG**GCGGCCGC**TTCAGTTCAGTGAAATTTATTGGAGA (NotI)
**p27 3' UTR**	CGGC**GATATC**ACAGCTCGAATTAAGAATATGTTTCC(EcoRV)	GCCG**GCGGCCGC**AATAGCTATGGAAGTTTTCTTTATTG (NotI)
**psiCHECk-2 (XhoI-NotI)**		
**Mouse MLL 3'UTR**	CGGC**CTCGAG**AGCTGTTCATCTTCCTGTGATGGAGA (XhoI)	GCCG**GCGGCCGC**TTTCCAGGGGGAAGCTGGGCAGGGA-3 (NotI)
**pGL3B (AatII-AgeI)**		
**MLL 3' UTR (1-3049)**	GCGC**GACGTC**AGCTGCTCTTCTCCCCCAGTGTTGGA (AatII)	GATC**ACCGGT**GGGGGATTCTTGGGAATGACCCATC (AgeI)
**pGL3B/P (KpnI-BglII)**		
**MLL 3' UTR**		
**(245-1143)**	CGGC**GGTACC**ACTGTTAGAAAGTGGGAATGGGGTCC (KpnI)	GCCG**AGATCT**TGTTCTGCCTTCAACTGGACACTGAC (BglII)
**(1143-245)**	CGGC**AGATCT**ACTGTTAGAAAGTGGGAATGGGGTCC (BglII)	GCCG**GGTACC**TGTTCTGCCTTCAACTGGACACTGAC (KpnI)

	Quick-Site Multi Site-Directed Mutagenesis Kit (Stratage)	
**MLL 3'UTR**		
**Δ(270-1140)**	ACTGTTAGAAAGTGGGAATGGGGTCACTAATCAGATTTCAAGGCCCACAAC	
**(1-1143)PAS**	TGGGGCCCCTCCAATTGTTTTGGCCGC**AATAAAA**TATCTTTATTTTCATTACATCTGTGTGTTGGTTTTTTGTGTGAACTGTTAGAAAGTGGGAATG	
**(1-1143)PAS MUT**	TGGGGCCCCTCCAATTGTTTTGGCCGC**CGATGCA**TATCTTTATTTTCATTACATCTGTGTGTTGGTTTTTTGTGTGAACTGTTAGAAAGTGGGAATG	
**(1-1143)SAP**	TGGGGCCCCTCCAATTGTTTTCACACAAAAAACCAACACACAGATGTAATGAAAATAAAGATA**TTTTATT**GCGGCCAACTGTTAGAAAGTGGGAATG	

	**Forward**	**Reverse**
**Gene expression**		
**Renilla**	CGGAAACTGGAGCCTGAGGA	AACCCAGGGTCGGACTCGAT
**b-actin**	CCTGGCACCCAGCACAAT	GGGCCGGACTCGTCATACT
**MLL Pair 5'**	GTAGTCCTACTCCTCTCT	GGTCTCTCTCTCTACTCT
**MLL Pair 3'**	CATTGATGCAGGTGAGATGG	GTGATTGATGAAGCGTGCAG
**AF9 Pair 5'**	TGCCGTGCAGGTGAAGCTGG	CCGGACCGCGTACGAACACC
**AF9 Pair 3'**	ACCCAGTCCTGCCAGCTCCA	CTGCGACTTCGGCTGCCTCC
**p27**	AGCGGAGCAATGCGCAGG	TCTTCTGAGGCCAGGCTTCT
**GAPDH**	CCCCACACACATGCACTTACC	CCTACTCCCAGGGCTTTGATT
