# Supplementary figures and images for: 3′UTR-Mediated Gene Silencing of the Mixed Lineage Leukemia (MLL) Gene
Source: PLoS One. 2011 Oct 5;6(10):e25449. doi: 10.1371/journal.pone.0025449 (PMC3187771; doi:10.1371/journal.pone.0025449)

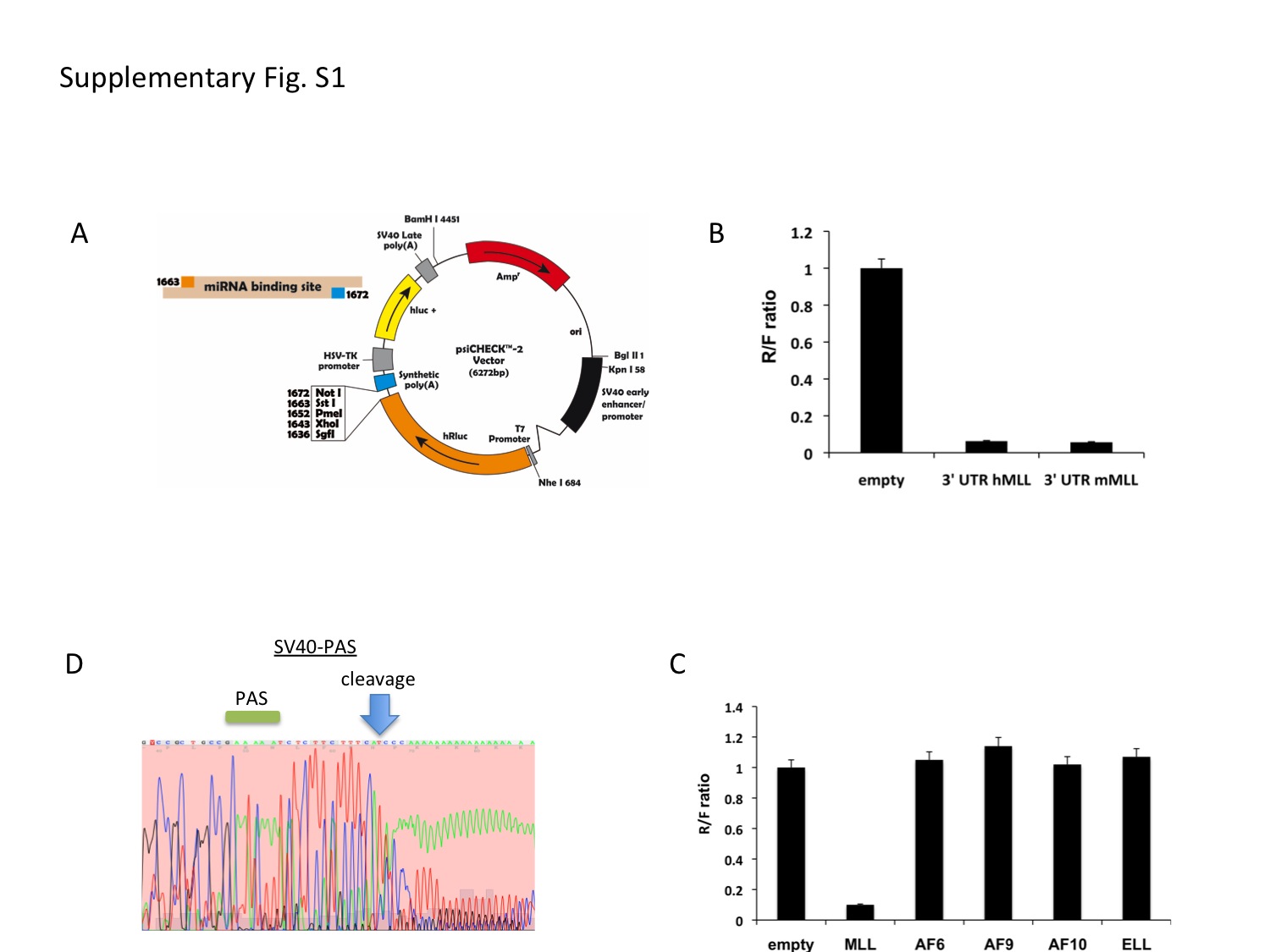

Supplement: Figure S1 — A, a schematic drawing of the psiCHECK reporter vector backbone used in this study. B, transient transfection of psiCHECK reporter constructs containing human and mouse MLL-3′UTRs in MCF-7 cells. C, transient transfection of psiCHECK reporter constructs containing MLL and main PG 3′UTRs. D, Cells were transfected with Ψ2-PAS-MLL-3′UTR and 3′end analysis was performed using RT with a P7-T25N oligo and PCR with P7 and a sequence specific primer. Sequence analysis of the PCR product shows the correct cleavage and polyadenylation induced by the SV40-PAS. (JPG) [file pone.0025449.s002.jpg]
