# Supplementary material for: 3′UTR-Mediated Gene Silencing of the Mixed Lineage Leukemia (MLL) Gene
Source: PLoS One. 2011 Oct 5;6(10):e25449. doi: 10.1371/journal.pone.0025449 (PMC3187771; doi:10.1371/journal.pone.0025449)
Supplement: Table S1 — Main clinical and molecular characteristics of cells lines used in MLL-PG and PG-MLL mRNA levels evaluation. MLL-PTD means MLL partial tandem duplication, AML-M2, AML-M4 and MLL-M5 refer to different subtypes of AML according to the French-American British (FAB) classification. T-ALL refers to T cell lynphocytic leukemia. (DOCX) [file pone.0025449.s003.docx]

**Supplementary Table S1**
