# Supplementary material for: 3′UTR-Mediated Gene Silencing of the Mixed Lineage Leukemia (MLL) Gene
Source: PLoS One. 2011 Oct 5;6(10):e25449. doi: 10.1371/journal.pone.0025449 (PMC3187771; doi:10.1371/journal.pone.0025449)
Supplement: Table S2 — Main clinical and molecular characteristics of patient samples are shown. Sex (M means male; F means female), AML-M2, AML-M4 and MLL-M5 refer to different subtypes of AML according to the French-American British (FAB) classification. ALL refers to acute lymphocytic leukemia and MDS to myelodysplastic syndrome. (DOCX) [file pone.0025449.s004.docx]

**Supplementary Table S2**
